# Mitochondrial Encephalopathy, Lactic Acidosis and Stroke-Like Episodes/Leigh Overlap Syndrome Due to Variant m.13513G>A in MT-ND5

**DOI:** 10.7759/cureus.24746

**Published:** 2022-05-05

**Authors:** Josef Finsterer, John Hayman

**Affiliations:** 1 Neurology, Neurology and Neurophysiology Centre, Vienna, AUT; 2 Clinical Pathology, The University of Melbourne, Melbourne, AUS

**Keywords:** hypotonia, genetics, respiratory failure, respiratory chain, mitochondrial disorder, mtdna, leigh syndrome

## Abstract

Mitochondrial disorders are caused due to variants in genes located on the mitochondrial DNA or the nuclear DNA. Here, we report a case with mitochondrial encephalopathy, lactic acidosis, and stroke-like episodes (MELAS)/Leigh overlap syndrome due to variant m.13513G>A in ND5. A 60-month-old female with a congenital, complex, multisystem phenotype was diagnosed with MELAS/Leigh overlap syndrome due to variant m.13513G>A in ND5*.* Brainstem involvement resulted in dysphagia, dysarthria, and respiratory failure with recurrent episodes of aspiration, respiratory insufficiency, desaturations, lack of respiratory drive, hypercapnia, and pneumonia. Treatment was symptomatic and included non-invasive ventilation, antibiotics, implantation of a percutaneous endoscopic gastrostomy, anti-seizure drugs, anti-dystonia medication, a cocktail of vitamins and antioxidants, and analgesics. Despite these measures, the outcome was poor as the patient died at the age of 62 months after being discharged to home palliative care. In summary, the m.13513G>A variant can manifest as MELAS/Leigh overlap syndrome with Leigh syndrome dominating. Because of brainstem involvement leading to respiratory dysfunction, dysarthria, and dysphagia, the outcome is poor, despite symptomatic measures.

## Introduction

Mitochondrial disorders (MIDs) are caused due to variants in genes located on the mitochondrial DNA (mtDNA) or the nuclear DNA (nDNA) [1]. mtDNA-located genes code for proteins, tRNAs, or rRNAs. One of the mtDNA genes coding for a protein is MT-ND5. MT-ND5 encodes for a complex-I subunit of the respiratory chain. One of the various variants in this gene is variant m.13513G>A. To our knowledge, at least 50 patients with this variant have been reported to date [2]. Due to the peculiarities of mitochondrial genetics, the phenotype of the m.13513G>A variant is variable. The variant manifests phenotypically as mitochondrial encephalopathy, lactic acidosis, and stroke-like episodes (MELAS) syndrome, Leigh syndrome, Leigh-like syndrome, progressive external ophthalmoplegia (PEO), MELAS/Leber’s hereditary optic neuropathy (LHON) overlap, LHON, or as MELAS/Leigh overlap syndrome [2]. To the best of our knowledge, only three patients with MELAS/Leigh overlap syndrome due to variant m.13513G>A have been reported till date. Here, we present a report on the fourth patient with this condition.

## Case presentation

The patient was a five-year-old female who was diagnosed with MELAS/Leigh overlap syndrome at the age of 1.5 years. The family history was unknown for MIDs. During pregnancy, polyhydramnion appeared from the 34th week of gestation. She was born at 39 weeks gestation by natural birth to non-consanguineous parents with a birth weight of 3000 g and Apgar scores of 9, 9 and 10, respectively. She soon presented with poor feeding (failure to thrive), anorexia, and facial hypotonia. From the age of six months, she suffered from recurring episodes of vomiting (after vaccination against the rotavirus). Hypotonia had become generalised since the eighth month of life. From the age of 11 months, the psychomotor development stopped (after a vaccination against influenza). After weaning from breast feeding at the age of 11 months, feeding problems worsened. She developed dystonic tremor from the age of 11 months. Bilateral ptosis, limb ataxia, and dysarthria developed from the age of 12 months. At the age of 18 months, a percutaneous endoscopic gastrostomy (PEG) was implanted after which the vomiting stopped. At the age of 20 months, she began to require assistance for walking, moved preferentially by crawling, and could eat only soft, mushy food. She started to use face cards for communication. At the age of 36 months, she underwent a genetic test that revealed the mtDNA variant m.13513G>A (p.D355N) in MT-ND5 with a heteroplasmy rate of 80% in blood lymphocytes. At the age of 54 months, she lost her ability to walk independently, respiratory dysfunction was recognised, and episodes of vomiting recurred. From the age of 59 months, she suffered from recurrent episodes of bradypnea, desaturation, respiratory acidosis, and shallow breathing, which have been attributed to brainstem involvement. Spasticity was noted from the age of 59 months. At 60 months of age, dystonic left upper limb movements appeared, spreading to the contralateral side, trunk, abdomen, and jaw.

**Figure 1:**
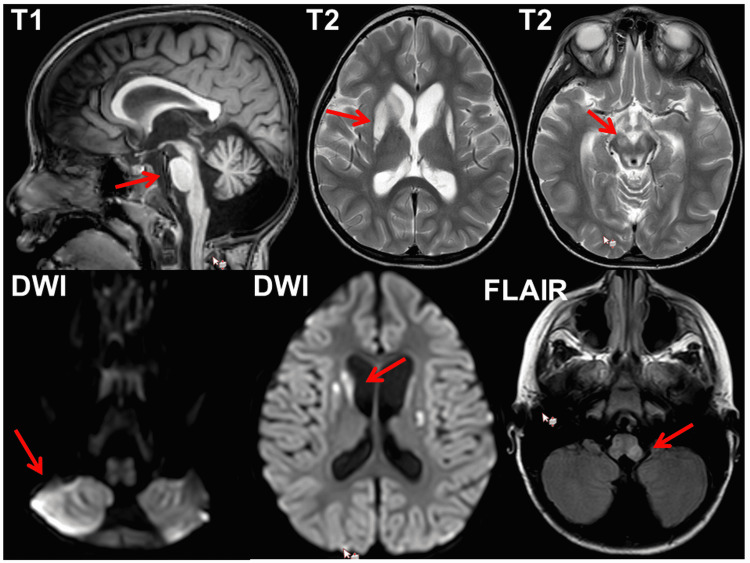
Cerebral MRI of the index patient at the age of 60 months showing cerebellar, pontine, and brainstem atrophy and callosal thinning on T1 (upper left), hyperintense basal ganglia lesions on T2 (upper middle), T2 hyperintense lesions in the midbrain (upper right), DWI hypereintense right cerebellar lesion (lower left), hyperintense basal ganglia lesions on DWI (lower middle), and hyperintense lesions in the medulla on TIRM (lower right) MRI, magnetic resonance imaging; DWI, diffusion-weighted imaging; FLAIR, fluid-attenuated inversion recovery; TIRM, turbo inversion recovery magnitude

**Figure 2:**
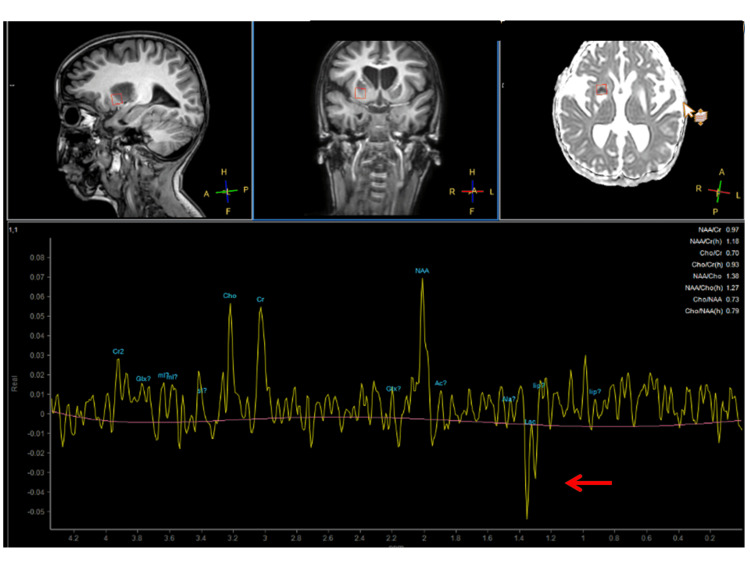
Cerebral MR spectrosocpy showing a lactate peak (arrow) in a sample of the basal ganglia MR, magnetic resonance

At the age of 60 months, she was hospitalised for respiratory failure with hypercapnia (pH 7.13) and aspiration pneumonia requiring temporary ventilatory support. During hospitalisation, she experienced recurrent focal and generalised seizures lasting for about 30 seconds that responded to midazolam. Her clinical neurologic exam revealed somnolence, hypertelorism, bilateral ptosis, divergent bulbs, absent pupillary reflexes, intermittent horizontal nystagmus, a reduced gag reflex, generalised muscle wasting, right hemispasm with exaggerated tendon reflexes, occasional right hemidystonia and tremor, and bilateral positive Babinski and Rossolimo signs. The EEG showed delta background activity (3-4.5 Hz) and intermittent beta-activity but no discharges typical of epilepsy. Brain magnetic resonance imaging (MRI) showed generalised cerebral atrophy (increased compared to a previous MRI), and T2/fluid-attenuated inversion recovery (FLAIR) hyperintensities in the basal ganglia, the midbrain, and medulla bilaterally, suggestive of Leigh syndrome (Figure 1). There was also a T2-hyperintense lesion in the right cerebellum that was hyperintense on diffusion-weighted imaging (DWI), suggesting a cerebellar stroke-like lesion (SLL) (Figure 1). MR spectroscopy revealed a lactate peak within the basal ganglia (Figure 2). ECG showed sinus tachycardia (110-160 bpm) and evidence of left ventricular hypertrophy. Her creatine kinase level was 133 U/l and the serum lactate level was 1.6 mmol/l. Since the seizures recurred, drug therapy was switched to levetiracetam and chloral hydrate. Her discharge medications included trihexyphenidyl (0.5 mg three times a day), chloral hydrate (17.5 ml/d), levetiracetam (280 mg/d, with an intent to taper down), tramadol, esomeprazole, and a vitamin cocktail. The patient was discharged home for palliative care, where she expired two months later.

## Discussion

The case presented here is interesting because of the overlap between phenotypic traits of MELAS and Leigh syndrome due to the ND5 variant m.13513G>A. Features of MELAS in the index patient included the suspected SLL in the left cerebellum, vomiting, myopathy, and suspected hypertrophic cardiomyopathy. Characteristics of Leigh syndrome included early onset, failure to thrive, developmental delay, bilateral basal ganglia lesions, dysphagia, dystonia, tremor, respiratory failure due to brainstem involvement, and generalised hypotonia. Progression of the illness after stressful events, in this case, inoculations and weaning, is also typical of this disorder. Features attributable to both syndromes included ptosis, epilepsy, spasticity, ataxia, dysarthria, and facial dysmorphism [3].

Patients carrying the ND5 variant n.13513G>A have been previously reported [4-20]. In a recent review, 50 patients with the m.13513G>A variant were recorded by the end of June 2021 [2]. The age group in this cohort ranged from birth to 63 years. The sex was male in 28 cases and female in 22 cases. The most common phenotypes were Leigh syndrome, MELAS, Leigh-like syndrome, MELAS/Leigh overlap syndrome, PEO, MELAS/LHON, and LHON. Some patients manifested with non-syndromic phenotypes, such as optic atrophy, cardiomyopathy, myopathy, strabismus, Wolff-Parkinson-White syndrome, nystagmus, seizures, ataxia, or fatal congenital acidosis [2]. Histochemical studies in patients undergoing muscle biopsy most commonly revealed NADH deficiency. The heteroplasmy rates were between 0% and 86%. The outcome was significantly worse in patients with Leigh syndrome than in patients with MELAS.

The three patients with MELAS/Leigh overlap syndrome reported so far were a 22-year-old female (patient 1), a 16-year-old male (patient 2), and a 13-year-old female (patient 3) [11]. Clinical and imaging features were similar to those of the index patient and characteristic of both MELAS and Leigh syndrome, but the clinical presentation differed depending on the number of MELAS or Leigh traits contributing to the fusion phenotype [11]. Patient 1 presented initially with features of MELAS followed at a later stage by features of Leigh syndrome. In patient 2, the imaging features of Leigh syndrome preceded those of MELAS. In patient 3, imaging features of MELAS and Leigh syndrome co-occurred. All three patients had undergone muscle biopsy and the heteroplasmy rate in the muscle ranged from 46% to 61%. Patients 1 and 3 showed ragged red fibers on muscle biopsy. Brain autopsy in patient 1 revealed infarct-like lesions in the cerebral cortex, basal ganglia, and brainstem [11].

A limitation of this study is that the parents or other first-degree relatives have not previously been subjected to genetic testing. Most likely, the causative m.13513G>A variant was sporadic, since both the parents and other first-degree relatives did not show phenotypic features of a MID. Also, heteroplasmy was only determined in blood lymphocytes. Other limitations are that the work-up for suspected cardiomyopathy was incomplete and that no muscle biopsy was performed to conduct biochemical investigations of the muscle homogenate to assess respiratory chain functions.

## Conclusions

The index case demonstrates that the m.13513G>A variant can manifest as MELAS/Leigh overlap syndrome with multisystem involvement. It shows that MELAS/Leigh overlap syndrome is a congenital, progressive MID manifesting with a complex phenotype, with features of Leigh syndrome prevailing; brainstem involvement is responsible for respiratory dysfunction, dysarthria, and dysphagia, all leading to a bad outcome. Therapy for MELAS/Leigh overlap syndrome is symptomatic and includes noninvasive ventilation, antibiotics, PEG placement, anti-seizure medications, medications for dystonia, cocktails of vitamins and antioxidants, and analgesics.
